# Fisetin Supplementation Attenuates Premature Vascular Aging Induced by Doxorubicin via Suppression of Cellular Senescence and Mitochondrial Oxidative Stress

**DOI:** 10.1111/acel.70535

**Published:** 2026-05-17

**Authors:** Mary A. Darrah, Sophia A. Mahoney, Ravinandan Venkatasubramanian, Nicholas S. VanDongen, Katelyn R. Ludwig, Douglas R. Seals, Matthew J. Rossman, Zachary S. Clayton

**Affiliations:** ^1^ University of Colorado Boulder Boulder Colorado USA; ^2^ University of Colorado Anschutz Medical Campus Aurora Colorado USA

**Keywords:** arterial function, cellular senescence, doxorubicin, premature aging, senolytics

## Abstract

The genotoxic agent doxorubicin induces premature vascular aging, defined by vascular endothelial dysfunction and aortic stiffening. Excess vascular cell senescence and the accompanying senescence‐associated secretory phenotype (SASP) are key mechanisms underlying doxorubicin‐induced vascular dysfunction, in part, by promoting excess mitochondrial oxidative stress, which reduces the bioavailability of the vasodilatory molecule nitric oxide (NO). In the present study, we assessed if the natural senolytic fisetin mitigates doxorubicin‐induced cellular senescence and the SASP to improve vascular function following doxorubicin administration and explored the underlying mechanisms. Young adult (6 months) mice were treated with doxorubicin, followed by oral, intermittent fisetin supplementation (100 mg/kg/day; 1 week on treatment–2 weeks off treatment–1 week on treatment). Vascular endothelial function, aortic stiffness, cellular senescence markers, SASP expression, NO bioavailability, and mitochondrial oxidative stress were assessed. Parallel experiments in human aortic endothelial cells were conducted to provide further mechanistic insight. Fisetin mitigated excess vascular cell senescence and the SASP in young mice administered doxorubicin and reversed doxorubicin‐induced endothelial dysfunction (*p* < 0.001) and aortic stiffening (*p* < 0.001), in part through suppression of excess cellular senescence, higher NO bioavailability, and lower mitochondrial oxidative stress. Modulation of the circulating SASP (plasma) also contributed to the observed vascular improvements with fisetin. In vitro, fisetin reduced cellular senescence in doxorubicin‐exposed endothelial cells, supporting isolated artery and in vivo observations. These findings identify oral intermittent fisetin supplementation as a promising therapeutic strategy for targeting excess cellular senescence to improve vascular function in settings of premature vascular aging.

## Graphical abstract

The natural senolytic Fisetin restores vascular function in a doxorubicin‐induced model of premature vascular aging via suppression of cellular senescence and the senescence‐associated secretory phenotype (SASP), thereby reducing mitochondrial oxidative stress and excess inflammation.

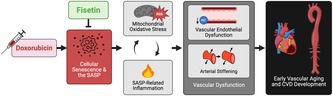

## 1 Introduction

Cardiovascular disease (CVD) is a major contributor to morbidity and mortality associated with premature aging (North and Sinclair 2012). Doxorubicin (Doxo), a potent inducer of cellular senescence and oxidative damage (Venkatasubramanian, Mahoney, et al. 2025), is widely used as a model compound to study mechanisms of premature aging (Feng et al. 2019). Exposure to Doxo in young adulthood leads to early onset of CVD, resembling those seen in chronologically aged individuals (Lee et al. 2020; Rawat et al. 2021). Thus, Doxo‐induced CV toxicity serves as a valuable framework for understanding how cellular and molecular processes of premature aging contribute to increased CVD risk and early mortality.

Elevated CVD risk with chronological aging is driven in part by impaired vascular endothelial function and aortic stiffening (Lakatta and Levy 2003). However, Doxo induces vascular dysfunction through similar mechanisms in young adulthood, reflecting a state of premature vascular aging (Parr et al. 2020; Luu et al. 2018; Clayton et al. 2021). Doxo‐mediated endothelial dysfunction, as shown by reduced endothelium‐dependent dilation (EDD), occurs as a result of reduced bioavailability of the vasodilatory molecule nitric oxide (NO) driven largely by excess reactive oxygen species (ROS), a key source of which is mitochondria (Clayton et al. 2020, Venkatasubramanian, Mahoney, et al. 2025). Doxo‐induced aortic stiffening, as indicated by increased aortic pulse wave velocity (PWV) and elastic modulus, is driven primarily by chronic inflammation (Venkatasubramanian, Darrah, et al. 2025; Clayton et al. 2021). As such, therapies that target these cellular/molecular processes hold promise for improving vascular function in models of premature aging, such as Doxo administration in young adulthood.

Cellular senescence is a multifaceted stress response that leads to a largely permanent cell cycle arrest (Campisi and d'Adda di Fagagna 2007). Excess senescent cells can accumulate in the vasculature and exacerbate mitochondrial ROS and inflammation in part by secreting—locally in the tissue microenvironment and/or into systemic circulation (e.g., the plasma)—numerous pro‐inflammatory cytokines collectively termed the senescence‐associated secretory phenotype (SASP) (Wang et al. 2024). As such, the SASP represents a key driver and measurable output of inflammation associated with cellular senescence. We have previously demonstrated that excess cellular senescence and the circulating SASP milieu directly contribute to premature vascular aging induced by Doxo, which established cellular senescence and the SASP as putative therapeutic targets for mitigating vascular dysfunction following Doxo administration (Venkatasubramanian, Mahoney, Hutton, et al. 2025; Venkatasubramanian, Darrah, et al. 2025). However, limited clinically translational strategies to reduce Doxo‐induced cellular senescence and the SASP exist.

Senolytics are compounds that selectively clear excess senescent cells and are emerging as a promising therapeutic approach in the context of chronological and premature aging (Mahoney et al. 2025). Fisetin is a flavonoid senolytic found in a number of commonly consumed fruits and vegetables with strong potential for clinical translation (Farsad‐Naeimi et al. 2018; Khan et al. 2013; Yousefzadeh et al. 2018; Wang et al. 2019). We recently showed that oral intermittent fisetin supplementation improves vascular function by reducing senescent cell burden in old mice (Mahoney et al. 2023, Mahoney et al. 2026). However, it is unknown if oral intermittent fisetin supplementation is effective for suppressing cellular senescence and vascular dysfunction induced by Doxo.

In the present study, we tested the hypotheses that (1) in human aortic endothelial cells, fisetin would reduce senescent cell burden following exposure to Doxo; (2) oral intermittent supplementation with fisetin would result in lower senescent cell burden in the arteries of mice following Doxo administration and improve vascular function; and (3) improvements in vascular function with oral intermittent fisetin supplementation would be mediated by suppression of excess cellular senescence, increased NO bioavailability, reduced mitochondrial ROS, and favorable modulation of the circulating SASP.

## 2 Methods

### 2.1 Cell Culture Experiments

Detailed description of the cell culture methods is available under Supporting Information. Briefly, human aortic endothelial cells (HAECs) were cultured under standard culture conditions. HAECs were treated with or without (control) 200 nM Doxo for 24 h (Demaria et al. 2017). Following the initial incubation, media was replaced and D, Mahonyoxo‐treated HAECs were treated with or without (control) increasing doses of fisetin (0.25, 0.5 and 1 μM) for 48 h (Mahoney et al. 2023).

### 2.2 Mouse Experiments

Detailed description of the animal methods is available under Supporting Information. Briefly, male and female p16‐3MR mice were bred and aged in our mouse colony at the University of Colorado Boulder. These mice carry a trimodal fusion protein (3MR) under the control of the p16 promoter which allows for selective genetic clearance of p16‐positive senescent cells by administering the antiviral agent ganciclovir (GCV) (Demaria et al. 2014).

For the intervention, treatment groups were matched for baseline body weight and aortic PWV. At 4 months of age, mice were assigned to receive either a single intraperitoneal injection of Sham (sterile saline) or Doxo (10 mg/kg in Sham). One week later, mice received treatment with either vehicle (10% Ethanol, 30% PEG400 and 60% Phosal 50 PG) or fisetin (100 mg/kg/day in vehicle), yielding four groups Sham‐Vehicle (*n* = 13), Sham‐Fisetin (*n* = 12), Doxo‐Vehicle (*n* = 11), and Doxo‐Fisetin (*n* = 14). Treatment was administered via oral gavage using an intermittent dosing paradigm consisting of 1 week on treatment–2 weeks off treatment–1 week on treatment (Figure 2A). Mice were sacrificed 1–2 weeks following the final dose to rule out any acute effects of the compound as the terminal half‐life of fisetin is ~3.1 h in plasma (Jo et al. 2016). Since we did not observe sex differences in any of the outcomes, male and female data were combined in the results.

### 2.3 Statistical Analyses

Detailed descriptions of all statistical analyses performed are provided in the Supplemental Methods. Data are presented as mean ± SEM in text, figures, and tables unless specified otherwise. Statistical significance was set to α = 0.05. All statistical analyses were performed using Prism, version 11 (GraphPad Software Inc., La Jolla, CA).

## 3 Results

### 3.1 Fisetin Reduces Doxo‐Induced Endothelial Cell Senescence In Vitro

#### 3.1.1 Senescence Burden in Cultured Endothelial Cells

**FIGURE 1:**
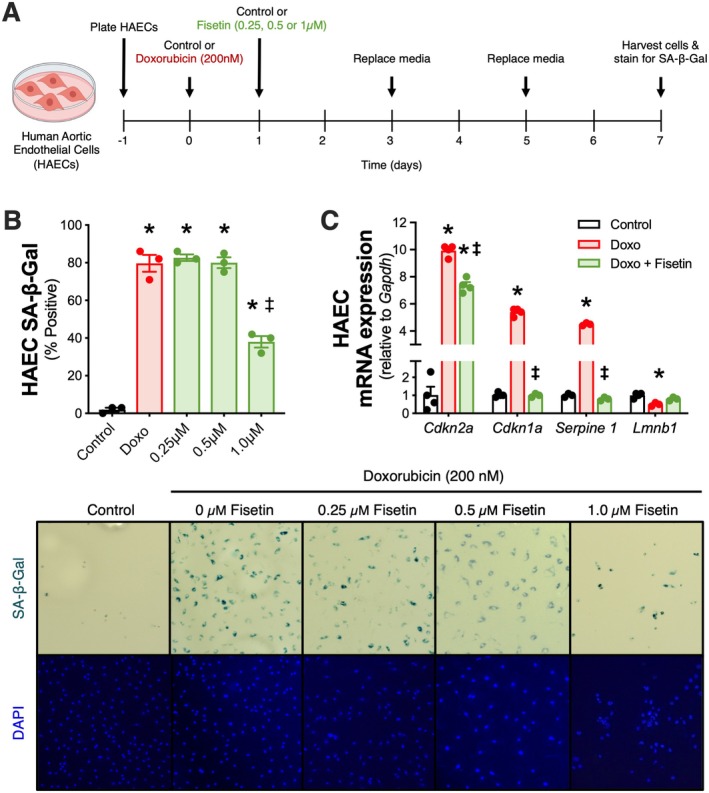
Treatment with fisetin reduces Doxorubicin (Doxo)‐induced cellular senescence in endothelial cells. Cultured human aortic endothelial cells (HAECs) were treated with Doxo (200 nM) or control for 24 h, treated with fisetin (0, 0.25, 0.5, or 1.0 μM) or control for 48 h, then recovered for 4 days (A). Senescence‐associated β‐galactosidase (SA‐β‐Gal) signal was measured in HAECs following treatments, representative images below (B). Gene expressions of cellular senescence biomarkers were measured in the HAECs following Doxo and 1.0 μM fisetin treatment (C). Data are mean ± SEM; *n* = 3–4/group; *p < 0.05 versus Control; ^‡^ *p* < 0.05 versus Doxo.

Initially, we performed in vitro cell culture experiments in HAECs to confirm the cellular senescence‐inducing effects of Doxo exposure (Figure 1A). We found that HAECs exposed to Doxo had ~80% higher senescence‐associated β‐galactosidase (SA‐β‐gal) signal, a canonical marker of cellular senescence (Kurz et al. 2000), relative to control HAECs (*p* < 0.001; Figure 1B). Next, to determine the extent to which fisetin could mitigate Doxo‐induced cellular senescence, HAECs were treated with increasing concentrations of fisetin (0.25, 0.5, or 1.0 μM) (Mahoney et al. 2023; Zhu et al. 2017) following Doxo exposure (Figure 1A). We found that while 0.25 and 0.5 μM fisetin treatment following Doxo exposure had a minimal effect on the SA‐β‐gal signal compared with Doxo exposure alone, 1.0 μM fisetin lowered Doxo‐induced senescent cell burden by ~50% (*p* < 0.001; Figure 1B), which is consistent with our previous results in cultured endothelial cells brought to senescence via replicative exhaustion (Mahoney et al. 2023, Mahoney et al. 2026). Importantly, 1.0 μM fisetin in vitro is a highly translational dose given that peak plasma levels of fisetin reach ~1.0 μM following oral ingestion of fisetin at a dose of 100 mg/kg/day in mice (Jo et al. 2016; Touil et al. 2011). As such, we selected 1.0 μM as our fisetin concentration for the remainder of our cell culture experiments.

To further establish the senolytic effects of fisetin in endothelial cells following Doxo exposure, we measured gene expression of established cellular senescence biomarkers in HAECs following Doxo exposure and subsequent fisetin treatment (1.0 μM). Cellular senescence is largely governed by two pathways that are responsible for irreversible cell cycle arrest: p16/Rb and p53/p21 (Tchkonia et al. 2013). Accordingly, we measured gene expression of *Cdkn2a* and *Cdkn1a*, which encode the cyclin‐dependent kinase inhibitor proteins, p16 and p21, respectively. Additionally, we measured cellular senescence biomarkers *Serpine1* (a gene that encodes PAI‐1, a plasminogen activator inhibitor sufficient for the induction of cellular senescence (Kortlever et al. 2006)) and *Lmnb1* (a gene that encodes Lamin B1, a contributor of nuclear wall stability shown to be reduced in senescent cells (González‐Gualda et al. 2021)). Compared with control HAECs, Doxo exposure increased expression of *Cdkn2a* by 9.9‐fold (*p* < 0.001), *Cdkn1a* by 5.4‐fold (*p* < 0.001), and *Serpine1* by 4.5‐fold (*p* < 0.001), and lowered *Lmnb1* by 0.5‐fold (*p* = 0.012; Figure 1C). Consistent with previously reported effects of fisetin for reducing senescent cell burden in endothelial cells (Mahoney et al. 2023; Zhu et al. 2017; Mahoney et al. 2026), 1.0 μM fisetin lowered *Cdkn2a* by 2.5‐fold (*p* < 0.001), *Cdkn1a* by 5.3‐fold (*p* < 0.001), and *Serpine1* by 3.8‐fold (*p* < 0.001), and tended to increase *Lmnb1* by 0.25‐fold (*p* = 0.186) in HAECs exposed to Doxo (Figure 1C).

Together, we observed higher senescent cell burden following Doxo exposure, demonstrated by both greater SA‐β‐gal signal and altered cellular senescence‐related gene expression in endothelial cells, which was favorably modulated back towards control levels when cells were treated with 1.0 μM fisetin. Thus, our in vitro experiments provide initial proof‐of‐principle efficacy for the use of fisetin to mitigate cellular senescence in arterial endothelial cells following Doxo exposure.

### 3.2 Animal Characteristics

**FIGURE 2:**
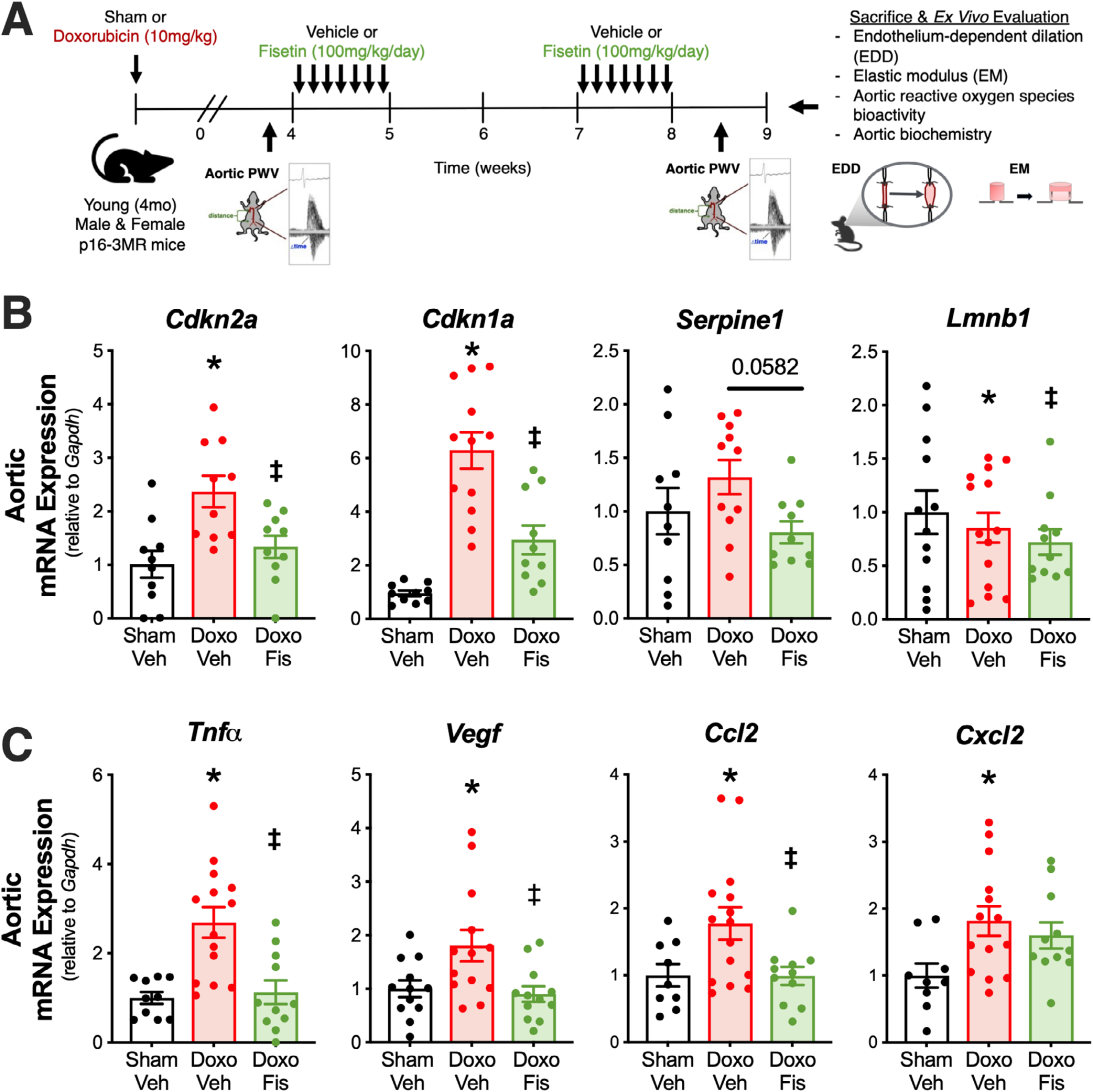
Oral intermittent fisetin supplementation reduces aortic expression of cellular senescence and senescence‐associated secretory phenotype (SASP) markers in Doxorubicin (Doxo)‐treated mice. Young adult (4 months) male and female p16‐3MR mice received a single intraperitoneal injection of sham (saline) or Doxo (10 mg/kg in saline). Four weeks later, animals were treated with vehicle (Veh; 10% ethanol, 30% PEG400, 60% Phosal 50 PG) or fisetin (Fis; 100 mg/kg/day in the vehicle solution) via oral gavage using an intermittent dosing paradigm (A). Aortic gene expression of cellular senescence (B) and SASP (C) markers in Sham‐Veh, Doxo‐Veh, and Doxo‐Fis treated groups. Data are mean ± SEM; *n* = 9–12/group; *p < 0.05 versus Sham‐Veh, ^‡^ *p* < 0.05 versus Doxo‐Veh.

To extend our findings of the senolytic effects of fisetin following Doxo exposure, we sought to assess cellular senescence in the vasculature of young adult mice that were administered Doxo and subsequently treated with fisetin (Figure 2A). Body mass, food intake, key organ mass, aorta characteristics, and frailty at the time of euthanasia for all treatment groups are reported in Table 1. Blood pressures, assessed following sham/Doxo administration and following the vehicle/fisetin intervention, are reported in Table 1. We found that compared with Sham‐Vehicle mice, Doxo‐Vehicle (*p* = 0.026) and Doxo‐Fisetin (*p* = 0.025) mice had lower body weight, despite no difference in food intake (Table 1). The observed lower body weight in animals that received Doxo was likely due to the associated lower fat mass in Doxo‐Vehicle (50% lower; *p* = 0.050 vs. Sham‐Vehicle) and Doxo‐Fisetin (32% lower; *p* = 0.237 vs. Sham‐Vehicle) mice, a phenotype previously observed following Doxo administration (Clayton et al. 2020; Elsea et al. 2015; Table 1). Moreover, we found that Doxo‐Vehicle mice scored higher on a clinically validated frailty index, an independent predictor of all‐cause and cardiovascular‐related mortality in humans (Court et al. 2025), compared with the Sham‐Vehicle treated animals (*p* = 0.031; Table 1), which is in line with previous literature showing greater frailty‐related phenotypes following Doxo administration (Cella et al. 2024). Doxo‐Fisetin animals tended to have lower (improved) frailty index scores compared with Doxo‐Vehicle mice (*p* = 0.068). Notably, frailty scores in the Doxo‐Fisetin animals were not different from the Sham‐Vehicle mice (*p* = 0.31; Table 1), indicating that oral intermittent fisetin supplementation may ameliorate the frailty‐promoting effects of Doxo. Otherwise, there were no major differences between groups in other listed organ masses, aorta characteristics, or blood pressures.

**TABLE 1:** Anthropometric, arterial, blood pressure and frailty characteristics of mice administered sham and Doxorubicin (Doxo) and subsequently treated with vehicle or fisetin.

	Sham‐vehicle	Sham‐fisetin	Doxo‐vehicle	Doxo‐fisetin
*n* (females; males)	13 (7; 6)	12 (7; 5)	11 (7; 4)	14 (9; 5)
Body weight (g)	26.9 ± 0.96	26.31 ± 1.56	23.53 ± 1.04*	23.50 ± 1.04*
Food intake (kcal/day)	10.8 ± 0.4	12.9 ± 0.5	12.4 ± 1.2	10.9 ± 0.6
Visceral adipose mass (mg)	392 ± 77	591 ± 133	194 ± 53*	267 ± 69*
Quadriceps mass (mg)	285 ± 10	283 ± 16	272 ± 14	286 ± 8
Heart mass (mg)	138 ± 4	148 ± 10	143 ± 6	132 ± 5
Left ventricular mass (mg)	81 ± 6	82 ± 10	76 ± 8	70 ± 4
Liver mass (g)	1.45 ± 0.07	1.46 ± 0.09	1.37 ± 0.08	1.37 ± 0.09
Spleen mass (mg)	70 ± 3	77 ± 5	68 ± 6	73 ± 5
Carotid artery (μM)				
Resting diameter	439 ± 4	433 ± 5	444 ± 6	439 ± 6
Maximal diameter	471 ± 5	469 ± 6	476 ± 6	472 ± 5
Aorta (μM)				
Diameter	665 ± 45	626 ± 20	618 ± 10	636 ± 11
Wall thickness	39 ± 2	45 ± 1	38 ± 2	44 ± 3
Systolic blood pressure (mm/Hg)				
Post Sham/doxo	102 ± 3	102 ± 2	99 ± 2	100 ± 5
Post vehicle/fisetin	108 ± 7	98 ± 3	106 ± 4	90 ± 5
Diastolic blood pressure (mm/Hg)				
Post Sham/doxo	75 ± 3	76 ± 3	72 ± 2	74 ± 5
Post vehicle/fisetin	83 ± 7	71 ± 4	71 ± 4	65 ± 5
Frailty index score	0.010 ± 0.004	0.017 ± 0.005	0.045 ± 0.014*	0.018 ± 0.006

*Note:* Data are mean ± SEM.

* *p* < 0.05 vs. Sham‐Vehicle.

### 3.3 Oral Fisetin Supplementation Reduces Vascular Cell Senescence and SASP Burden in Mice Administered Doxo

#### 3.3.1 Vascular Cell Senescence Burden

To translate our findings that fisetin ameliorates the elevated senescent cell burden with Doxo in cultured endothelial cells, we assessed the gene expression of canonical senescence markers in arteries from animals that were administered Doxo and treated with fisetin. We observed higher expression of *Cdkn2a* (2.3‐fold, *p* < 0.001) and *Cdkn1a* (6.3‐fold, *p* < 0.001) in aortas from Doxo‐Vehicle mice compared with the Sham‐Vehicle animals (Figure 2B). The senescence‐promoting effects of Doxo were diminished following oral intermittent fisetin supplementation, such that compared with the Doxo‐Vehicle animals, the Doxo‐Fisetin group had lower aortic expression of *Cdkn2a* by 2‐fold (*p* = 0.029) and *Cdkn1a* by 3.4‐fold (*p* < 0.001; Figure 2B). We did not observe any major differences across groups in expression of *Serpine1* (Doxo‐Vehicle vs. Sham‐Vehicle: *p* = 0.358; Doxo‐Vehicle vs. Doxo‐Fisetin: *p* = 0.058) or *Lmnb1* (Doxo‐Vehicle vs. Sham‐Vehicle: *p* = 0.774; Doxo‐Vehicle vs. Doxo‐Fisetin: *p* = 0.760; Figure 2B).

#### 3.3.2 Vascular SASP Burden

We next assessed gene expression of common SASP markers in arteries from our four groups of animals, which included inflammatory cytokines, chemokines, and growth factors (Saul et al. 2022). Of these factors, we found that compared with the Sham‐Vehicle mice, Doxo‐Vehicle mice had higher expression of *Tnfα* (2.7‐fold, *p* < 0.001), *Vegf* (1.9‐fold, *p* = 0.003), *Ccl2* (1.8‐fold, *p* = 0.009), and *Cxcl2* (1.7‐fold, *p* = 0.025; Figure 2C). However, the Doxo‐Fisetin animals exhibited lower expression of *Tnfα* (2.6‐fold, *p* < 0.001), *Vegf* (1.9‐fold, *p* = 0.001), and *Ccl2* (1.8‐fold, *p* = 0.006), but not *Cxcl2* (0.2‐fold, *p* = 0.452) compared with the Doxo‐Vehicle group (Figure 2C). There were no differences in *Tnfα, Vegf*, or *Ccl2* expression between the Doxo‐Fisetin treatment group and the Sham‐Vehicle treatment group, suggesting that oral intermittent fisetin supplementation was largely able to abolish the increase in these SASP markers elicited by Doxo administration.

In combination, these data in arteries extend our findings in cultured aortic endothelial cells, suggesting that Doxo administration results in higher senescent cell and SASP burden in the vasculature of mice, and oral intermittent fisetin supplementation following Doxo can lower these markers back to Sham‐Vehicle control levels.

### 3.4 Oral Intermittent Fisetin Supplementation Ameliorates Doxo‐Induced Endothelial Dysfunction

#### 3.4.1 Endothelial Function

**FIGURE 3:**
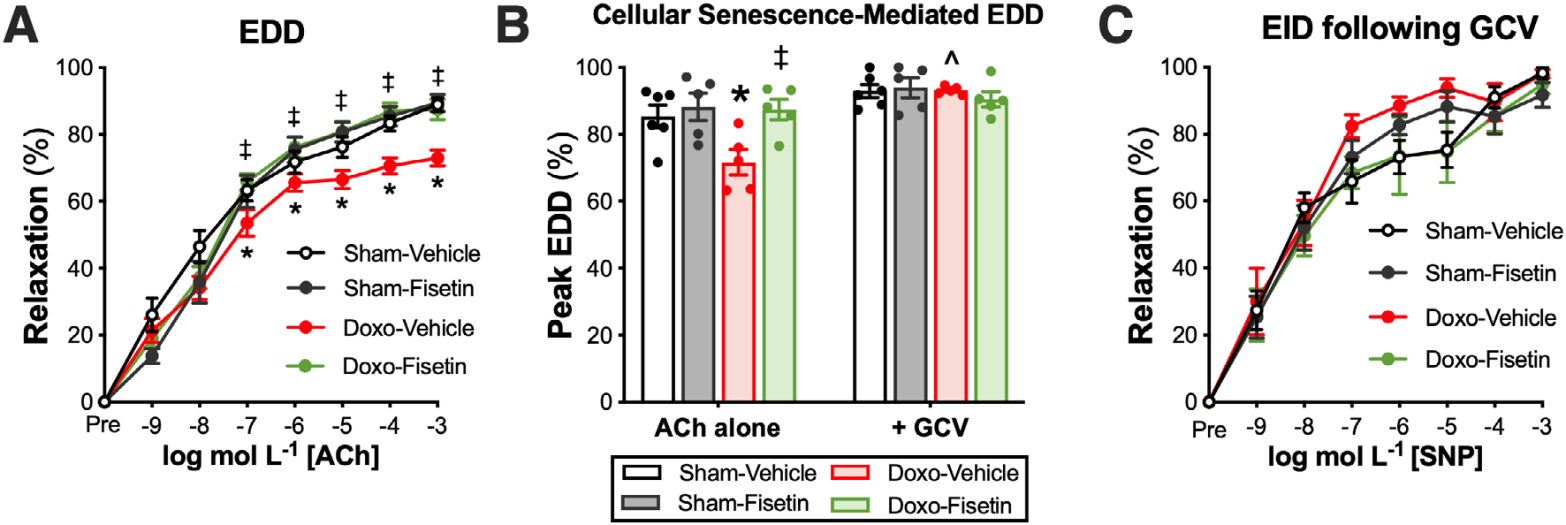
Oral intermittent fisetin supplementation improves endothelial dysfunction caused by Doxorubicin (Doxo) administration by suppressing excess cellular senescence. Endothelium‐dependent dilation (EDD) to increasing doses of acetylcholine (ACh) (A). Peak EDD following ex vivo ganciclovir (GCV, 5 μM) incubation compared with incubation with ACh alone (B). Endothelium‐independent dilation (EID) to the exogenous nitric oxide donor, sodium nitroprusside (SNP) following GCV incubation (C). Data are mean ± SEM; *n* = 5–7/group; *p < 0.05 versus Sham‐Vehicle, ^‡^ *p* < 0.05 versus Doxo‐Vehicle, ^*p* < 0.05 versus Doxo‐Vehicle ACh alone.

To assess the impact of Doxo administration and oral intermittent fisetin supplementation on vascular endothelial function, we assessed carotid artery EDD to increasing doses of acetylcholine (ACh). Mice from the Doxo‐Vehicle group exhibited lower peak EDD relative to Sham‐Vehicle mice (Doxo‐Vehicle, 73% ± 2% vs. Sham‐Vehicle, 89% ± 2%, *p* = 0.003), indicating Doxo administration impaired endothelial function (Figure 3A,B), which is consistent with what we have previously reported (Venkatasubramanian, Mahoney, et al. 2025; Clayton et al. 2020). Oral intermittent fisetin supplementation restored peak EDD, as evidenced by higher EDD in the Doxo‐Fisetin mice compared with the Doxo‐Vehicle animals (Doxo‐Fisetin, 89% ± 3%, *p* < 0.001), and similar EDD to the Sham‐Vehicle (*p* = 0.972) and Sham‐Fisetin (89% ± 3%, *p* = 0.924) groups (Figure 3A,B). There were no differences in peak EDD between the Sham‐Vehicle and Sham‐Fisetin animals (*p* = 0.891), suggesting no effects of oral intermittent fisetin supplementation on endothelial function in young adult, healthy mice (Figure 3A,B).

#### 3.4.2 Cellular Senescence‐Mediated Suppression of Endothelial Function

To investigate the role of cellular senescence as an underlying mechanism of endothelial dysfunction with Doxo administration and improved endothelial function with oral intermittent fisetin supplementation, we used a subset of mice from each of the four study groups and incubated their carotid arteries ex vivo with GCV (to clear senescent cells ex vivo) prior to assessing EDD with ACh. We then compared EDD following GCV incubation to the carotid arteries incubated with ACh alone. Importantly, ex vivo GCV incubation in arteries from p16‐3MR mice is an established experimental approach to interrogate the role of excess cellular senescence in modulating endothelial function (Mahoney et al. 2023). Compared with EDD with ACh alone, the addition of GCV increased EDD in the Doxo‐Vehicle group (ACh alone, 72% ± 4% vs. with GCV, 93% ± 1%, *p* = 0.005), suggesting excessive cellular senescence contributes to endothelial dysfunction following Doxo administration (Figure 3C). The addition of GCV did not alter peak EDD of the Doxo‐Fisetin (ACh alone, 87% ± 3% vs. with GCV, 91% ± 2%, *p* = 0.239), Sham‐Vehicle (ACh alone, 85% ± 3% vs. with GCV, 93% ± 2%, *p* = 0.082), or Sham‐Fisetin (ACh alone, 88% ± 4% vs. with GCV, 94% ± 3%, *p* = 0.311) animals (Figure 3C). The lack of group differences in EDD following GCV incubation in these groups (i.e., groups without excess senescent cell burden) suggests that oral intermittent fisetin supplementation selectively restores EDD following Doxo administration, in part, by clearing excess senescent cells. Next, to determine if GCV influenced smooth muscle function, we assessed endothelium‐independent dilation (EID) following the GCV incubation. No differences between groups were observed in peak EID (Sham‐Vehicle, 98% ± 2% vs. Sham‐Vehicle, 92% ± 4% vs. Doxo‐Vehicle, 98% ± 1% vs. Doxo‐Fisetin, 95% ± 3%, *p* = 0.171), signifying that Doxo‐induced cellular senescence impairs vasodilation in an endothelium‐specific manner (Figure 3D). Taken together, these data indicate that a mechanism by which oral intermittent fisetin supplementation improved vascular endothelial function following Doxo administration was by reducing excessive cellular senescence.

#### 3.4.3 NO Bioavailability

**FIGURE 4:**
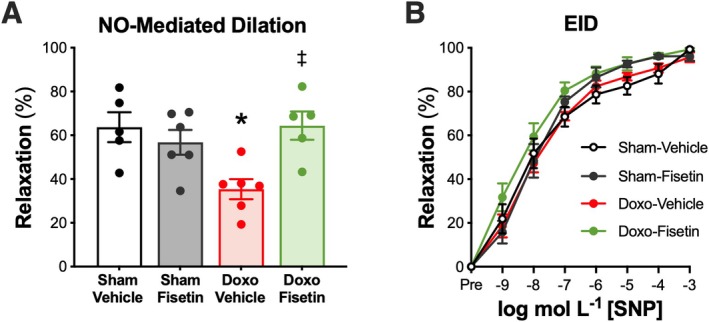
Oral intermittent fisetin supplementation improves endothelial dysfunction resulting from Doxorubicin (Doxo) administration by increasing nitric oxide (NO) bioavailability. NO–mediated, endothelium‐dependent dilation was calculated after ex vivo incubation with the NO synthase inhibitor L‐NAME as follows: Maximal dilation to acetylcholine (ACh) minus maximal dilation to ACh in the presence of L‐NAME (A). Endothelium‐independent dilation (EID) to the exogenous NO donor, sodium nitroprusside (SNP) (B). Data are mean ± SEM; *n* = 5–12/group; *p < 0.05 versus Sham‐Vehicle; ^*p* < 0.05 versus – L‐NAME; ^‡^ *p* < 0.05 versus Doxo‐Vehicle.

We have previously shown that lower endothelial function with Doxo administration occurs as a result of reduced NO bioavailability (Venkatasubramanian, Mahoney, et al. 2025; Clayton et al. 2020); whereas oral intermittent fisetin supplementation can enhance NO bioavailability and improve endothelial function in other settings of cellular senescence‐mediated endothelial dysfunction (i.e., aging) (Mahoney et al. 2023; Clayton et al. 2023). Thus, in a subset of mice, we sought to determine the effects of oral intermittent fisetin supplementation on NO bioavailability following Doxo administration. To accomplish this, we assessed ACh‐induced EDD with and without the presence of the NO synthase inhibitor L‐NAME. We first established that mice from the Doxo‐Vehicle group had lower NO‐mediated EDD compared with the Sham‐Vehicle group (Doxo‐Vehicle, 35% ± 5% vs. Sham‐Vehicle, 64% ± 7%, *p* = 0.006), confirming our previous observations that reduced NO bioavailability underlies the impairment in EDD with Doxo (Figure 4A). Mice in the Doxo‐Fisetin group had higher peak NO‐mediated dilation (64% ± 6%) as compared with the Doxo‐Vehicle mice (*p* = 0.005), suggesting that oral intermittent fisetin supplementation restored NO bioavailability following Doxo administration (Figure 4A). There were no differences in NO‐mediated EDD between the Doxo‐Fisetin group and both the Sham‐Vehicle (*p* = 0.944) and Sham‐Fisetin (57% ± 6%, *p* = 0.397) groups, demonstrating full restoration of NO‐mediated EDD with oral intermittent fisetin supplementation following Doxo administration (Figure 4A). Further, we did not observe a difference in NO‐mediated EDD between the Sham‐Vehicle and Sham‐Fisetin groups (*p* = 0.450), inferring minimal effects of oral intermittent fisetin supplementation on NO bioavailability in young adult, healthy animals (Figure 4A). Together, these data suggest that the improvements observed in endothelial function with oral intermittent fisetin supplementation following Doxo administration are mediated, in part, by enhanced NO bioavailability. Finally, to determine if these improvements in endothelial function were specific to the endothelium and not due to enhanced smooth muscle cell sensitivity to NO, we assessed EID and found no differences between groups (Sham‐Vehicle, 99% ± 1% vs. Sham‐Fisetin, 96% ± 2% vs. Doxo‐Vehicle, 96% ± 2% vs. Doxo‐Fisetin, 99% ± 1%, *p* = 0.275), signifying differences in EDD occurred in an endothelium‐specific manner (Figure 4B). Collectively, these data suggest that Doxo impairs endothelial function in mice in a NO‐dependent manner, consistent with our previous findings (Clayton et al. 2020), and oral intermittent fisetin supplementation following Doxo administration restores endothelial function by increasing NO bioavailability back to healthy, young levels.

### 3.5 Oral Intermittent Fisetin Supplementation Restores Endothelial Function Following Doxo Administration by Doxo Suppressing Excessive Mitochondrial Oxidative Stress

#### 3.5.1 Vascular Mitochondrial Oxidative Stress

**FIGURE 5:**
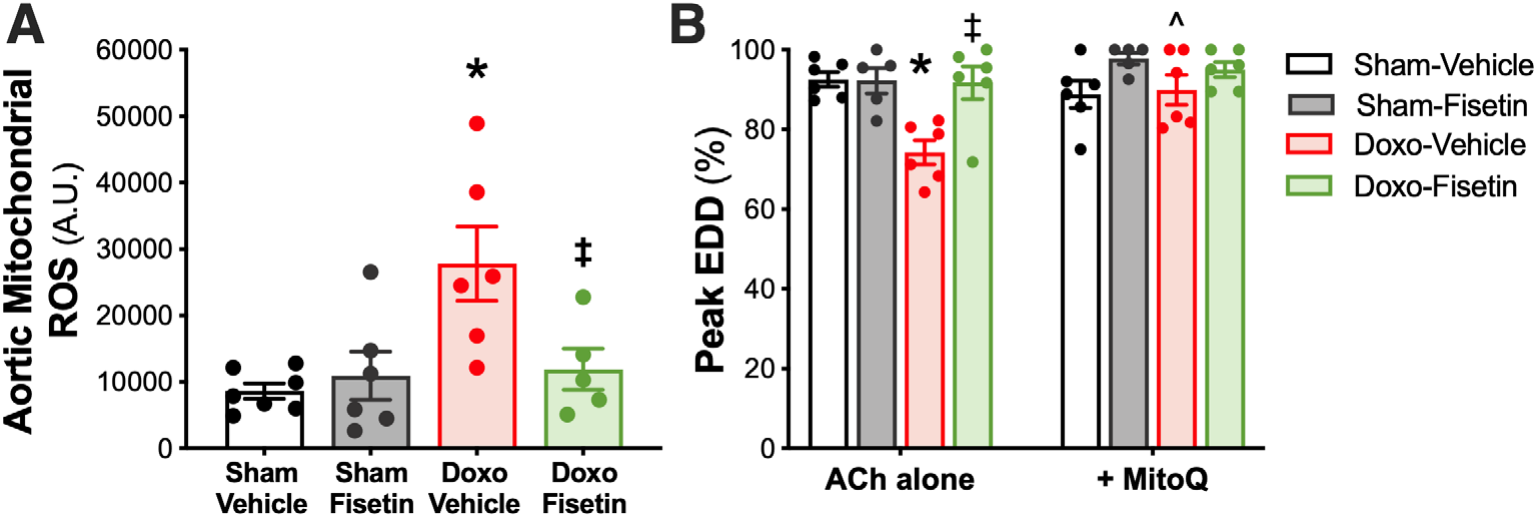
Oral intermittent fisetin supplementation restores the Doxorubicin (Doxo)‐induced decrease in endothelial function by lowering mitochondrial oxidative stress. Aortic mitochondrial reactive oxygen species (ROS) levels of the Sham‐Vehicle (Veh), Sham‐Fisetin, Doxo‐Veh, and Doxo‐Fisetin treated groups (A). Peak endothelium‐dependent dilation (EDD) with acetylcholine (ACh) alone versus ACh with MitoQ, a mitochondrial‐targeted antioxidant (B). Data are mean ± SEM; *n* = 5–7/group; *p < 0.05 versus Sham‐Vehicle, ^‡^ *p* < 0.05 versus Doxo‐Vehicle, ^*p* < 0.05 versus Doxo‐Vehicle ACh alone.

We have previously shown that Doxo administration increases mitochondrial ROS‐related oxidative stress in the vasculature, which is directly implicated in Doxo‐induced endothelial dysfunction (Clayton et al. 2020). Moreover, we have demonstrated that the senolytic‐mediated improvement in endothelial function with oral intermittent fisetin supplementation was associated with lower mitochondrial ROS bioactivity with aging (Mahoney et al. 2023). Thus, considering our previous observations in the present study that oral intermittent fisetin supplementation improved endothelial function following Doxo administration by reducing cellular senescence, we next sought to determine the influence of oral intermittent fisetin supplementation on mitochondrial ROS bioactivity following Doxo administration. To accomplish this, we first assessed aortic mitochondrial ROS bioactivity and found that the Doxo‐Vehicle mice had 3.2‐fold higher aortic mitochondrial ROS bioactivity compared with Sham‐Vehicle mice (Doxo‐Vehicle, 27,840 ± 5590 arbitrary units [AU] vs. Sham‐Vehicle, 8636 ± 1165 AU, *p* = 0.004), indicative of greater mitochondrial ROS‐related oxidative stress (Figure 5A). Oral intermittent fisetin supplementation ameliorated this difference, as aortic mitochondrial ROS bioactivity was lower in Doxo‐Fisetin mice compared with Doxo‐Vehicle animals (Doxo‐Fisetin, 11,920 ± 3101 AU, *p* = 0.044), but not different compared with Sham‐Vehicle (*p* = 0.288) or Sham‐Fisetin (10,943 ± 3625 AU, *p* = 0.846; Figure 5A).

#### 3.5.2 Excessive Mitochondrial Oxidative Stress‐Mediated Suppression of Endothelial Function

To determine if oral intermittent fisetin supplementation following Doxo administration improved endothelial function via reduction of excessive mitochondrial ROS bioactivity resulting from Doxo administration, we assessed EDD in a subset of animals with and without ex vivo addition of MitoQ (a mitochondrial‐targeted antioxidant (Powell et al. 2015)) prior to assessing EDD. Compared with ACh alone, the addition of MitoQ increased peak EDD from the Doxo‐Vehicle mice (ACh alone, 74% ± 3% vs. with MitoQ, 90% ± 4%, *p* = 0.005), confirming our previous findings that excessive mitochondrial ROS bioactivity contributes to endothelial dysfunction following Doxo administration (Clayton et al. 2020; Figure 5B). The addition of MitoQ to the vessel perfusate did not alter peak EDD compared with ACh alone in the Doxo‐Fisetin group (ACh alone, 92% ± 4% vs. with MitoQ, 95% ± 2%, *p* = 0.529), signifying that the effects of Doxo on excessive mitochondrial ROS‐related suppression of EDD were ameliorated with oral intermittent fisetin supplementation (Figure 5B). Moreover, no additional improvements in EDD following MitoQ incubation were observed in the Sham‐Vehicle (ACh alone, 92% ± 4% vs. with MitoQ, 95% ± 2%, *p* = 0.395) or Sham‐Fisetin (ACh alone, 92% ± 3% vs. with MitoQ, 98% ± 1%, *p* = 0.241) groups suggesting the effects of MitoQ were specific to Doxo (Figure 5B). Treatment differences among groups in peak EDD were abolished upon incubation with MitoQ (*p* = 0.138), suggesting that Doxo impaired endothelial function via excessive mitochondrial ROS, which was ameliorated with oral intermittent fisetin supplementation (Figure 5B). Together, these data indicate that oral intermittent fisetin supplementation enhances endothelial function in mice administered with Doxo by ameliorating excessive mitochondrial ROS.

### 3.6 Oral Intermittent Fisetin Supplementation Reduces Doxo‐Induced Aortic Stiffening by Clearing Excess Senescent Cells

#### 3.6.1 Aortic Stiffness

**FIGURE 6:**
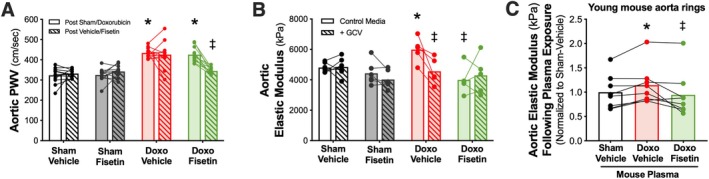
Oral intermittent fisetin supplementation reverses Doxorubicin (Doxo)‐induced aortic stiffness by suppressing excess cellular senescence and modulating the circulating milieu. In vivo aortic pulse wave velocity (PWV) of treatment groups following sham/Doxo administration (solid) and intermittent vehicle/fisetin supplementation (hatched) (A). Aortic elastic modulus of isolated aorta rings from Sham‐Vehicle, Sham‐Fisetin, Doxo‐Vehicle, and Doxo‐Fisetin treated groups following 48 h incubation in control media alone (solid) or with ganciclovir (GCV, 5 μM; hatched), a genetic senescent cell‐clearing compound in the p16‐3MR mouse model (B). Paired aortic elastic modulus of young, intervention‐naïve mice following plasma exposure from the Sham‐Vehicle, Doxo‐Vehicle, and Doxo‐Fisetin treated mice (C). Data are mean ± SEM; *n* = 5–13/group; *p < 0.05 versus Sham‐Vehicle, ^‡^ *p* < 0.05 versus Doxo‐Vehicle, ^*p* < 0.05 versus Post‐Doxo.

Given that oral intermittent fisetin supplementation restored endothelial function following Doxo administration, we next sought to determine another key manifestation of Doxo‐induced vascular dysfunction, aortic stiffening, using the reference standard in vivo approach, PWV (Venkatasubramanian, Darrah, et al. 2025; Clayton et al. 2021). We found that aortic PWV was higher in animals following Doxo administration as compared with the sham‐treated animals (Doxo‐Vehicle, 426 ± 20 cm/s vs. Sham‐Vehicle, 332 ± 5 cm/s, *p* < 0.001; and Doxo‐Fisetin, 426 ± 10 cm/s vs. Sham‐Fisetin, 325 ± 9 cm/s, *p* < 0.001; Figure 6A). However, the Doxo‐induced increase in aortic stiffness was reversed following oral intermittent fisetin supplementation (Doxo‐Fisetin, post‐fisetin, 345 ± 5 cm/s, *p* = 0.001 vs. pre‐fisetin; Figure 6A). There were no changes to aortic PWV following oral intermittent vehicle or fisetin supplementation in the sham control animals (*p* = 0.293), indicating that fisetin had no effect on aortic stiffness in healthy young adult animals (Figure 6A). These observations occurred in the absence of any changes in blood pressure, as blood pressure was not different among groups (Table 1).

#### 3.6.2 Cellular Senescence‐Mediated Aortic Stiffening

After establishing the reversal of Doxo‐induced aortic stiffening by oral intermittent fisetin supplementation, we sought to determine if the fisetin‐mediated reduction in aortic stiffness following Doxo administration was directly due to decreased senescent cell burden in arteries. To do so, aortic rings from treated animals were incubated in either control media alone or with 5 μM GCV to genetically clear excess senescent cells in the p16‐3MR mouse model, an approach we have previously used to assess the influence of cellular senescence in mediating age‐related aortic stiffening ex vivo (Mahoney et al. 2023). We found that when incubated in the control media alone, aorta rings from Doxo‐Vehicle mice exhibited higher elastic modulus as compared with Sham‐Vehicle mice (Doxo‐Vehicle, 5991 ± 362 kPa vs. Sham‐Vehicle, 4813 ± 133 kPa, *p* = 0.009), suggesting that Doxo administration promoted higher intrinsic mechanical wall stiffness (Figure 6B), which is consistent with our previous observations (Clayton et al. 2021). Aorta rings from Doxo‐Fisetin animals exhibited lower aortic elastic modulus compared with the Doxo‐Vehicle group when incubated in the control media alone (Doxo‐Fisetin, 3996 ± 415 kPa; *p* = 0.010 vs. Doxo‐Vehicle), suggesting that oral intermittent fisetin supplementation mitigated the increased aortic intrinsic mechanical wall stiffness resulting from Doxo administration (Figure 6B). There were no differences in elastic modulus between the Sham‐Vehicle and Sham‐Fisetin treated groups (Sham‐Fisetin, 4429 ± 314 kPa, *p* = 0.286; Figure 6B). Upon addition of GCV to the vessel incubation media, aorta rings from the Doxo‐Vehicle group exhibited lower elastic modulus when exposed to GCV as compared with incubation in the control media alone (4563 ± 385 kPa, *p* = 0.023), suggesting that excess cellular senescence mediated the higher aortic stiffness observed in the Doxo‐Vehicle mice (Figure 6B). However, we did not see any within‐group changes in elastic modulus in the aorta rings from Sham‐Vehicle (4848 ± 251 kPa, *p* = 0.902), Sham‐Fisetin (4021 ± 241 kPa, *p* = 0.128), or Doxo‐Fisetin (4298 ± 557 kPa, *p* = 0.610) groups compared with control media alone (Figure 6B), suggesting a lack of excess senescent cell‐mediated aortic stiffening in these groups. Further, all group differences were eliminated when the aorta rings were incubated in GCV (*p* = 0.397), implicating that fisetin mechanistically reverses Doxo‐induced aortic stiffening, in part, by suppressing excess senescent cells (Figure 6B).

### 3.7 Oral Intermittent Fisetin Supplementation Mitigates Vascular Dysfunction Resulting From Doxo Administration via Influence of the Circulating SASP Milieu

#### 3.7.1 Circulating SASP Milieu in Mediating Vascular Function

Next, we sought to determine the putative role of the circulating SASP milieu in mediating vascular function with Doxo administration and oral intermittent fisetin supplementation. Importantly, the circulating milieu is consistently in direct contact with the arterial wall, and the circulating SASP milieu has been implicated in age‐related (Clayton et al. 2023) and Doxo‐induced (Venkatasubramanian, Darrah, et al. 2025) aortic stiffening. Thus, to determine the influence of doxo administration and the effects of oral intermittent fisetin supplementation on circulating SASP milieu‐mediated changes in vascular function, we obtained aorta rings from young adult (6 months) intervention‐naïve female and male p16‐3MR mice and incubated them for 48 h in standard cell culture media containing 10% plasma (a pool of the circulating milieu) from the sex‐matched mice in the Sham‐Vehicle, Doxo‐Vehicle, and Doxo‐Fisetin groups. Given that no functional differences were observed between the two sham‐treated groups, only plasma from the Sham‐Vehicle group was used. Following plasma exposure, aortic elastic modulus was measured relative to the Sham‐Vehicle group. Plasma from the Doxo‐Vehicle mice evoked an increase in aortic elastic modulus compared with Sham‐Vehicle plasma (Doxo‐Vehicle, 1.14 ± 0.14 vs. Sham‐Vehicle, 1.0 ± 0.12, *p* = 0.015), suggesting the circulating SASP milieu from the Doxo‐Vehicle mice contributed to aortic stiffening (Figure 6C). Plasma from Doxo‐Fisetin mice transduced lower aortic stiffness compared with Doxo‐Vehicle (Doxo‐Fisetin, 0.946 ± 0.17, *p* = 0.035) and had values similar to aortas exposed to Sham‐Vehicle plasma (*p* = 0.655), suggesting an amelioration of circulating SASP milieu‐mediated vascular dysfunction (Figure 6C). In combination, these data support previous results that the circulating SASP milieu contributes to aortic stiffness following Doxo administration (Venkatasubramanian, Darrah, et al. 2025), and oral intermittent fisetin supplementation likely improves vascular function following Doxo administration, in part, by favorably modulating the circulating SASP milieu.

## 4 Discussion

Doxo administration in young adulthood induces a state of premature vascular aging, characterized by an increase in cellular senescence burden in the vasculature and a state of vascular dysfunction that occurs as a result of excessive cellular senescence (Venkatasubramanian, Mahoney, et al. 2025; Venkatasubramanian, Darrah, et al. 2025). Our findings provide the first evidence supporting the efficacy of oral fisetin supplementation for reducing senescent cell burden and reversing cellular senescence‐induced vascular dysfunction following Doxo administration. We first determined that fisetin mitigated Doxo‐induced cellular senescence in cultured endothelial cells. We then used a mouse model to determine that oral intermittent fisetin supplementation following Doxo administration reduced vascular senescent cell and SASP burden, ameliorated Doxo‐induced endothelial dysfunction, and reversed aortic stiffening. Together, these findings demonstrate that oral intermittent fisetin supplementation may be a promising therapeutic strategy for restoring vascular function and mitigating features of premature vascular aging induced by Doxo.

### 4.1 Fisetin as a Therapeutic Strategy to Mitigate Premature Vascular Aging

Doxo administration represents a well‐established experimental model of stress‐induced vascular senescence that recapitulates several molecular and functional features associated with vascular aging. Although this model does not fully reproduce the complexity of all vascular aging processes, Doxo administration has been shown to replicate key features of age‐related vascular dysfunction, including endothelial dysfunction, aortic stiffening, and excess vascular senescence (Shamoon et al. 2022; Abdeahad et al. 2025; Venkatasubramanian, Mahoney, Hutton, et al. 2025).

As such, findings from this study may be extended to other forms of premature aging that demonstrate high levels of stress‐induced cellular senescence burden. Select groups that may experience early vascular aging and a greater senescence burden due to detrimental lifestyle choices, genotoxic drugs, genetic mutations, or disease progression (Venkatasubramanian, Mahoney, et al. 2025; Venkatasubramanian, Darrah, et al. 2025; Mahoney et al. 2025). For example, states of early vascular aging induced by medications essential for disease management like antiretroviral therapy have also been shown to induce premature vascular dysfunction (Kanmogne 2024). Further, comorbidities, such as type 2 diabetes, arthritis, dyslipidemia, polycystic ovary disease, hormonal changes, and pregnancy‐related conditions such as preeclampsia, are all associated with premature vascular aging (Henry et al. 2004; Meireles et al. 2024; Paradisi et al. 2001; Powe et al. 2011; Corrigan et al. 2015; Moreau and Hildreth 2014). Thus, fisetin may be a promising therapeutic for reversing premature aging phenotypes in these populations by decreasing endothelial cell senescence and improving NO bioavailability, thereby improving vascular function.

### 4.2 Fisetin Reduces Doxo‐Induced Vascular Cell Senescence

Vascular senescent cells contribute to a myriad of vascular diseases including atherosclerosis (Minamino and Komuro 2007), arterial pulmonary hypertension (Noureddine et al. 2011), and aortic aneurysms (Lu et al. 2021), all of which are anteceded by vascular dysfunction (Mahoney et al. 2025). Considering that Doxo increases senescent cell burden in the vasculature, we sought to leverage senolytic therapy as a therapeutic strategy to reduce vascular senescent cell burden (Mahoney et al. 2025). Although several senolytics have been discovered to‐date, the natural senolytic fisetin is considered one of the safest and more accessible for human clinical translation (Farsad‐Naeimi et al. 2018; Khan et al. 2013; Yousefzadeh et al. 2018; Wang et al. 2019) and has been shown to reduce vascular cell senescence and improve vascular function (i.e., in the setting of advanced age (Mahoney et al. 2023)). Our findings demonstrated that fisetin decreased Doxo‐induced senescent endothelial cell burden in cultured human endothelial cells, and this suppression of cellular senescence with fisetin was similar to previous observations with fisetin administration in endothelial cells brought to senescence via replicative exhaustion (Mahoney et al. 2023), suggesting broad senolytic properties of fisetin across various cell senescence‐inducing stressors.

The vasculature is considered to be among the most susceptible tissues to undergoing senescence in models of premature aging (Yousefzadeh et al. 2020). Likewise, in the preclinical setting, we observed a robust vascular response in cellular senescence and SASP burden following Doxo administration. Fisetin treatment ameliorated Doxo‐induced senescent cell burden back to basal levels in the vasculature to a similar extent as previously observed in chronological aging models (Venkatasubramanian, Mahoney, et al. 2025), indicating that fisetin may have wide‐ranging senolytic effects against multiple stressors that trigger cellular senescence.

### 4.3 Fisetin Ameliorates Doxo‐Induced Vascular Endothelial Dysfunction

Impaired vascular endothelial function, a key manifestation of vascular dysfunction, contributes to the development of CVDs such as atherosclerosis (Hadi et al. 2005). Doxo administration has been associated with reduced vascular endothelial function in human cancer survivors (Parr et al. 2020; Terwoord et al. 2022), but clinical mechanistic insight is limited. One established underlying mechanism contributing to lower endothelial function with Doxo is excess cellular senescence, but clinically viable senolytic therapies have yet to be established. In the current study, we first demonstrated that Doxo impaired endothelial function, consistent with previous findings (Venkatasubramanian, Mahoney, et al. 2025), and that oral intermittent fisetin supplementation ameliorated Doxo‐induced endothelial dysfunction. We leveraged the p16‐3MR mouse model to demonstrate that cellular senescence is the underlying mechanism by which fisetin improves endothelial function following the impairments induced with Doxo administration.

A key mechanism by which Doxo exerts its adverse effects is through mitochondrial dysfunction (Clayton et al. 2020). Excess mitochondrial ROS sequesters NO to reduce its bioavailability and impair endothelial function (Clayton et al. 2020). Importantly, both mitochondrial dysfunction and diminished NO bioavailability are hallmarks of vascular cell senescence (Hayashi et al. 2006). Previously, we have shown that oral intermittent fisetin supplementation can improve age‐related endothelial function by enhancing NO bioavailability and suppressing excessive mitochondrial ROS bioactivity (Mahoney et al. 2023). In this extension of previous work, we showed that the same dosing paradigm of fisetin effectively ameliorated endothelial dysfunction by improving NO bioavailability and ameliorating mitochondrial ROS‐related suppression of endothelial function in the context of Doxo administration.

### 4.4 Fisetin Ameliorates Doxo‐Induced Large Elastic Artery Stiffening: Potential Role of the Circulating SASP Milieu

Large elastic artery (e.g., aorta) stiffening, an independent risk factor for cognitive impairment (Hirasawa et al. 2022), kidney disease (Tian et al. 2024) and vision loss (Sato et al. 2006), represents another key manifestation of vascular dysfunction. We have previously shown Doxo administration directly induces aortic stiffening in young adult mice (Clayton et al. 2021). Moreover, Doxo administration in patients with cancer results in higher aortic stiffness compared with age‐matched healthy counterparts (Yersal et al. 2018), but the mechanisms underlying clinical aortic stiffening remain unknown. We recently showed that cellular senescence is an underlying mechanism of Doxo‐induced aortic stiffening and demonstrated proof‐of‐principle efficacy for the use of senolytic therapy to mitigate aortic stiffening following Doxo administration (Venkatasubramanian, Darrah, et al. 2025). As such, in the present study we sought to target cellular senescence with oral intermittent fisetin supplementation to reduce Doxo‐induced aortic stiffening, as we have previously shown that a similar fisetin dosing paradigm could effectively lower aortic stiffness in old mice (Mahoney et al. 2023). We found that oral intermittent fisetin supplementation reduced aortic stiffness following Doxo administration, which occurred in part by direct suppression of cellular senescence. Together, these findings provide evidence that fisetin may be a viable therapeutic strategy for reducing aortic stiffening in setting of premature aging.

The circulating SASP milieu is a collection of bioactive molecules in the bloodstream that are released from senescent cells and are in direct and frequent contact with the arterial wall (Mahoney et al. 2026). Importantly, the pathogenic effects of the SASP are mediated by the collective actions of multiple factors, rather than changes in single factors (Anaforoglu et al. 2012; Ohtani 2022). Our previous findings indicate that the circulating SASP milieu mediates vascular dysfunction following Doxo administration (Venkatasubramanian, Darrah, et al. 2025). In the current study, we determined that oral intermittent fisetin supplementation mitigates Doxo‐induced vascular dysfunction in part by modulating the circulating SASP milieu.

### 4.5 Protective Effects of Fisetin on Doxo‐Induced Frailty and Metabolic Alterations

Frailty is an independent predictor of CVD‐related and all‐cause mortality and a key phenotype of premature aging (Court et al. 2025; Cella et al. 2024). Doxo administration in cancer patients yields greater risk for frailty‐related conditions, and consequently, increased risk for premature morbidity and mortality as a result of developing CVD (Court et al. 2025; Guida et al. 2019; Fulop et al. 2010; Ness and Wogksch 2020). Heightened frailty is often accompanied by reductions in body weight and adipose tissue mass in cancer patients administered with Doxo chemotherapy compared with age‐ and sex‐matched healthy counterparts (Delaney et al. 2021; Extermann et al. 2017). In the present study, using a validated clinical frailty index (Whitehead et al. 2014), we provide evidence that oral intermittent fisetin supplementation may be a safe and effective strategy for reducing frailty following Doxo administration. Additionally, Doxo‐treated mice in the present study exhibited significantly lower body weight and visceral adipose tissue mass compared with sham controls, likely reflecting broader adverse systemic metabolic changes. Adipose tissue is an important source of systemic inflammatory signaling; therefore, reductions in adipose tissue mass may influence vascular function, independent of Doxo‐toxicity (Berg and Scherer 2005). In the present study, the observed effect of the circulating milieu on arterial stiffening may partially reflect the systemic physiological adaptations to Doxo exposure, including reduced adiposity, in addition to the direct Doxo‐induced vascular injury. Despite the reductions in body mass and adiposity observed in both the groups administered Doxo, fisetin supplementation partially mitigated the adverse effects of the circulating milieu. These findings suggest that fisetin's beneficial effects may occur independently of gross changes in adiposity, highlighting its promise as a therapeutic strategy to mitigate Doxo‐induced vascular dysfunction and frailty.

## 5 Study Limitations

While Doxo is a valuable model of premature vascular aging, it may not be a universal model of all forms of early vascular aging. Doxo induces acute and supraphysiologic stress, whereas other premature aging‐inducing processes (e.g., metabolic or inflammatory) are chronic and multifactorial. Doxo primarily causes mitochondrial and nuclear DNA damage, while metabolic aging also involves lipid toxicity and insulin resistance. Finally, cellular injury with Doxo may not fully capture immune or metabolic dysregulation aspects of diseases like diabetes or obesity. Importantly, several clinical trials are investigating various frailty‐associated outcomes following intermittent fisetin supplementation in states of premature aging including chemotherapy (NCT06819254, NCT04733534, NCT05595499, and NCT06113016), COVID‐19 (NCT94476953, NCT04771611, NCT 04537299), and CVD (NCT06399809). Further, clinical trials in healthy mid‐life and older adults investigating the impact of fisetin supplementation in improving vascular function by decreasing cellular senescence are currently underway (NCT06133634). If these ongoing clinical trials demonstrate safety, tolerability and efficacy of fisetin supplementation to target cellular senescence and improve physiological function, these results, in combination with the results from the present study, would provide scientific premise for oral intermittent fisetin supplementation to improve vascular function in groups with premature vascular aging.

## 6 Conclusions

The current study provides evidence for the efficacy of a natural senolytic, fisetin, in improving vascular function following administration of Doxo, by reversing vascular endothelial dysfunction and aortic stiffening through suppression of excess cellular senescence, improved NO bioavailability, and favorable modulation of the circulating milieu. Though there are several clinical trials testing fisetin supplementation in states of premature aging, there are no studies to date investigating the effects of senolytic treatment for improving vascular function in groups with premature vascular aging. Thus, our preclinical findings provide necessary proof‐of‐principle evidence of efficacy supporting the need for future studies examining the potential senolytic properties of fisetin supplementation in improving vascular function in people with premature vascular aging.

## Author Contributions

M.A.D., S.A.M. and Z.S.C. are responsible for drafting the manuscript. M.A.D., S.A.M., D.R.S., M.J.R., Z.S.C. are responsible for study conception and design. M.A.D., S.M., R.V., N.S.V., K.R.L., Z.S.C. are responsible for carrying out the study. All authors approve of this final version of the manuscript.

## Funding

This work was supported by F31 HL165885 (to S.A.M.), R21 AG078408 (to D.R.S. & Z.S.C.), AHA 23CDA1056582 (to M.J.R.), and K99 HL159241 (to Z.S.C.).

## Conflicts of Interest

The authors declare no conflicts of interest.

## Supporting information


**Table S1:** Primers.

## Data Availability

The data that support the findings of this study are available from the corresponding author upon reasonable request.

## References

[acel70535-bib-0001] Abdeahad, H. , D. G. Moreno , S. I. Bloom , L. Norman , L. A. Lesniewski , and A. J. Donato . 2025. “MitoQ Reduces Senescence Burden in Doxorubicin‐Treated Endothelial Cells by Reducing Mitochondrial ROS and DNA Damage.” American Journal of Physiology. Heart and Circulatory Physiology 329, no. 5: H1154–H1161. 10.1152/ajpheart.00568.2025. 41026856 PMC12758503

[acel70535-bib-0002] Anaforoglu, I. , K. Ersoy , and E. Algun . 2012. “Parathyroid Adenoma With Coeliac Disease: Primary or Quaternary Hyperparathyroidism?” Endokrynologia Polska 63, no. 1: 56–58. 22378099

[acel70535-bib-0003] Berg, A. H. , and P. E. Scherer . 2005. “Adipose Tissue, Inflammation, and Cardiovascular Disease.” Circulation Research 96, no. 9: 939–949. 10.1161/01.RES.0000163635.62927.34. 15890981

[acel70535-bib-0004] Campisi, J. , and F. d'Adda di Fagagna . 2007. “Cellular Senescence: When Bad Things Happen to Good Cells.” Nature Reviews. Molecular Cell Biology 8, no. 9: 729–740. 10.1038/nrm2233. 17667954

[acel70535-bib-0005] Cella, P. S. , R. L. N. de Matos , P. C. Marinello , et al. 2024. “Doxorubicin Causes Cachexia, Sarcopenia, and Frailty Characteristics in Mice.” PLoS One 19, no. 4: e0301379. 10.1371/journal.pone.0301379. 38648220 PMC11034664

[acel70535-bib-0007] Clayton, Z. S. , V. E. Brunt , D. A. Hutton , et al. 2020. “Doxorubicin‐Induced Oxidative Stress and Endothelial Dysfunction in Conduit Arteries Is Prevented by Mitochondrial‐Specific Antioxidant Treatment.” JACC CardioOncology 2, no. 3: 475–488. 10.1016/j.jaccao.2020.06.010. 33073250 PMC7561020

[acel70535-bib-0006] Clayton, Z. S. , V. E. Brunt , D. A. Hutton , et al. 2021. “Tumor Necrosis Factor Alpha‐Mediated Inflammation and Remodeling of the Extracellular Matrix Underlies Aortic Stiffening Induced by the Common Chemotherapeutic Agent Doxorubicin.” Hypertension 77, no. 5: 1581–1590. 10.1161/HYPERTENSIONAHA.120.16759. 33719511 PMC8035245

[acel70535-bib-0008] Clayton, Z. S. , M. J. Rossman , S. A. Mahoney , et al. 2023. “Cellular Senescence Contributes to Large Elastic Artery Stiffening and Endothelial Dysfunction With Aging: Amelioration With Senolytic Treatment.” Hypertension 80, no. 10: 2072–2087. 10.1161/HYPERTENSIONAHA.123.21392. 37593877 PMC10530538

[acel70535-bib-0009] Corrigan, F. E. , I. Al Mheid , D. J. Eapen , et al. 2015. “Low Testosterone in Men Predicts Impaired Arterial Elasticity and Microvascular Function.” International Journal of Cardiology 194: 94–99. 10.1016/j.ijcard.2015.05.065. 26022684 PMC9135451

[acel70535-bib-0010] Court, T. , N. Capkova , A. Pająk , A. Tamosiunas , M. Bobák , and H. Pikhart . 2025. “Frailty Index Is an Independent Predictor of All‐Cause and Cardiovascular Mortality in Eastern Europe: A Multicentre Cohort Study.” Journal of Epidemiology and Community Health 79, no. 1: 56–63. 10.1136/jech-2023-221761. PMC11671974 39181708

[acel70535-bib-0011] Delaney, A. , C. R. Howell , K. R. Krull , et al. 2021. “Progression of Frailty in Survivors of Childhood Cancer: A St. Jude Lifetime Cohort Report.” JNCI Journal of the National Cancer Institute 113, no. 10: 1415–1421. 10.1093/jnci/djab033. 33720359 PMC8633430

[acel70535-bib-0012] Demaria, M. , N. Ohtani , S. A. Youssef , et al. 2014. “An Essential Role for Senescent Cells in Optimal Wound Healing Through Secretion of PDGF‐AA.” Developmental Cell 31, no. 6: 722–733. 10.1016/j.devcel.2014.11.012. 25499914 PMC4349629

[acel70535-bib-0013] Demaria, M. , M. N. O'Leary , J. Chang , et al. 2017. “Cellular Senescence Promotes Adverse Effects of Chemotherapy and Cancer Relapse.” Cancer Discovery 7, no. 2: 165–176. 10.1158/2159-8290.CD-16-0241. 27979832 PMC5296251

[acel70535-bib-0014] Elsea, C. R. , J. A. Kneiss , and L. J. Wood . 2015. “Induction of IL‐6 by Cytotoxic Chemotherapy Is Associated With Loss of Lean Body and Fat Mass in Tumor‐Free Female Mice.” Biological Research for Nursing 17, no. 5: 549–557. 10.1177/1099800414558087. 25406461 PMC4469616

[acel70535-bib-0015] Extermann, M. , C. Leeuwenburgh , L. Samiian , et al. 2017. “Impact of Chemotherapy on Medium‐Term Physical Function and Activity of Older Breast Cancer Survivors, and Associated Biomarkers.” Journal of Geriatric Oncology 8, no. 1: 69–75. 10.1016/j.jgo.2016.09.004. 27743848 PMC5299045

[acel70535-bib-0016] Farsad‐Naeimi, A. , M. Alizadeh , A. Esfahani , and E. Darvish Aminabad . 2018. “Effect of Fisetin Supplementation on Inflammatory Factors and Matrix Metalloproteinase Enzymes in Colorectal Cancer Patients.” Food & Function 9, no. 4: 2025–2031. 10.1039/c7fo01898c. 29541713

[acel70535-bib-0017] Feng, M. , J. Kim , K. Field , C. Reid , I. Chatzistamou , and M. Shim . 2019. “Aspirin Ameliorates the Long‐Term Adverse Effects of Doxorubicin Through Suppression of Cellular Senescence.” FASEB Bioadvances 1, no. 9: 579–590. 10.1096/fba.2019-00041. 32123852 PMC6996307

[acel70535-bib-0018] Fulop, T. , A. Larbi , J. M. Witkowski , et al. 2010. “Aging, Frailty and Age‐Related Diseases.” Biogerontology 11, no. 5: 547–563. 10.1007/s10522-010-9287-2. 20559726

[acel70535-bib-0019] González‐Gualda, E. , A. G. Baker , L. Fruk , and D. Muñoz‐Espín . 2021. “A Guide to Assessing Cellular Senescence In Vitro and In Vivo.” FEBS Journal 288, no. 1: 56–80. 10.1111/febs.15570. 32961620

[acel70535-bib-0020] Guida, J. L. , T. A. Ahles , D. Belsky , et al. 2019. “Measuring Aging and Identifying Aging Phenotypes in Cancer Survivors.” JNCI Journal of the National Cancer Institute 111, no. 12: 1245–1254. 10.1093/jnci/djz136. 31321426 PMC7962788

[acel70535-bib-0021] Hadi, H. A. , C. S. Carr , and J. Al Suwaidi . 2005. “Endothelial Dysfunction: Cardiovascular Risk Factors, Therapy, and Outcome.” Vascular Health and Risk Management 1, no. 3: 183–198. 17319104 PMC1993955

[acel70535-bib-0022] Hayashi, T. , H. Matsui‐Hirai , A. Miyazaki‐Akita , et al. 2006. “Endothelial Cellular Senescence Is Inhibited by Nitric Oxide: Implications in Atherosclerosis Associated With Menopause and Diabetes.” Proceedings of the National Academy of Sciences 103, no. 45: 17018–17023. 10.1073/pnas.0607873103. PMC1629003 17075048

[acel70535-bib-0023] Henry, R. M. A. , I. Ferreira , P. J. Kostense , et al. 2004. “Type 2 Diabetes Is Associated With Impaired Endothelium‐Dependent, Flow‐Mediated Dilation, but Impaired Glucose Metabolism Is Not: The Hoorn Study.” Atherosclerosis 174, no. 1: 49–56. 10.1016/j.atherosclerosis.2004.01.002. 15135250

[acel70535-bib-0024] Hirasawa, A. , K. Nagai , T. Miyazawa , et al. 2022. “Relationship Between Arterial Stiffness and Cognitive Function in Outpatients With Dementia and Mild Cognitive Impairment Compared With Community Residents Without Dementia.” Journal of Geriatric Cardiology 19, no. 8: 594–602. 10.11909/j.issn.1671-5411.2022.08.002. 36339473 PMC9630006

[acel70535-bib-0025] Jo, J. H. , J. J. Jo , J. M. Lee , and S. Lee . 2016. “Identification of Absolute Conversion to Geraldol From Fisetin and Pharmacokinetics in Mouse.” Journal of Chromatography B 1038: 95–100. 10.1016/j.jchromb.2016.10.034. 27810278

[acel70535-bib-0026] Kanmogne, G. D. 2024. “HIV Infection, Antiretroviral Drugs, and the Vascular Endothelium.” Cells 13, no. 8: 672. 10.3390/cells13080672. 38667287 PMC11048826

[acel70535-bib-0027] Khan, N. , D. N. Syed , N. Ahmad , and H. Mukhtar . 2013. “Fisetin: A Dietary Antioxidant for Health Promotion.” Antioxidants & Redox Signaling 19, no. 2: 151–162. 10.1089/ars.2012.4901. 23121441 PMC3689181

[acel70535-bib-0028] Kortlever, R. M. , P. J. Higgins , and R. Bernards . 2006. “Plasminogen Activator Inhibitor‐1 Is a Critical Downstream Target of p53 in the Induction of Replicative Senescence.” Nature Cell Biology 8, no. 8: 877–884. 10.1038/ncb1448. 16862142 PMC2954492

[acel70535-bib-0029] Kurz, D. J. , S. Decary , Y. Hong , and J. D. Erusalimsky . 2000. “Senescence‐Associated β‐Galactosidase Reflects an Increase in Lysosomal Mass During Replicative Ageing of Human Endothelial Cells.” Journal of Cell Science 113, no. 20: 3613–3622. 10.1242/jcs.113.20.3613. 11017877

[acel70535-bib-0065] Lakatta, E. G. , and D. Levy . 2003. “Arterial and Cardiac Aging: Major Shareholders in Cardiovascular Disease Enterprises.” Circulation 107, no. 1: 139–146. 10.1161/01.cir.0000048892.83521.58. 12515756

[acel70535-bib-0030] Lee, S. F. , M. A. Luque‐Fernandez , Y. H. Chen , et al. 2020. “Doxorubicin and Subsequent Risk of Cardiovascular Diseases Among Survivors of Diffuse Large B‐Cell Lymphoma in Hong Kong.” Blood Advances 4, no. 20: 5107–5117. 10.1182/bloodadvances.2020002737. 33085755 PMC7594396

[acel70535-bib-0031] Lu, H. , W. Du , L. Ren , et al. 2021. “Vascular Smooth Muscle Cells in Aortic Aneurysm: From Genetics to Mechanisms.” Journal of the American Heart Association 10, no. 24: e023601. 10.1161/JAHA.121.023601. 34796717 PMC9075263

[acel70535-bib-0032] Luu, A. Z. , B. Chowdhury , M. Al‐Omran , H. Teoh , D. A. Hess , and S. Verma . 2018. “Role of Endothelium in Doxorubicin‐Induced Cardiomyopathy.” JACC: Basic to Translational Science 3, no. 6: 861–870. 10.1016/j.jacbts.2018.06.005. 30623145 PMC6314956

[acel70535-bib-0033] Mahoney, S. A. , S. I. Bloom , D. R. Seals , A. J. Donato , M. J. Rossman , and Z. S. Clayton . 2025. “Mechanisms of Cellular Senescence‐Induced Vascular Aging: Evidence of Senotherapeutic Strategies.” Journal of Cardiovascular Aging 5, no. 2: 6. 10.20517/jca.2024.31. 40936799 PMC12422706

[acel70535-bib-0064] Mahoney, S. A. , K. Mazan‐Mamczarz , D. Tsitsipatis , et al. 2026. “Senolytic Treatment With Fisetin Reverses Age‐Related Endothelial Dysfunction Partially Mediated by SASP Factor CXCL12.” Aging Cell 25, no. 5. 10.1111/acel.70500. PMC13103471 42021544

[acel70535-bib-0034] Mahoney, S. A. , R. Venkatasubramanian , M. A. Darrah , et al. 2023. “Intermittent Supplementation With Fisetin Improves Arterial Function in Old Mice by Decreasing Cellular Senescence.” Aging Cell 23: e14060. 10.1111/acel.14060. 38062873 PMC10928570

[acel70535-bib-0035] Meireles, K. , T. Peçanha , A. J. Pinto , et al. 2024. “Improved Vascular Health Linked to Increased Physical Activity Levels and Reduced Sedentary Behavior in Rheumatoid Arthritis.” American Journal of Physiology‐Heart and Circulatory Physiology 327, no. 6: H1590–H1598. 10.1152/ajpheart.00640.2024. 39485295 PMC11684939

[acel70535-bib-0036] Minamino, T. , and I. Komuro . 2007. “Vascular Cell Senescence: Contribution to Atherosclerosis.” Circulation Research 100, no. 1: 15–26. 10.1161/01.RES.0000256837.40544.4a. 17204661

[acel70535-bib-0037] Moreau, K. L. , and K. L. Hildreth . 2014. “Vascular Aging Across the Menopause Transition in Healthy Women.” Advances in Vascular Medicine 2014: 1–12. 10.1155/2014/204390. PMC4433172 25984561

[acel70535-bib-0038] Ness, K. K. , and M. D. Wogksch . 2020. “Frailty and Aging in Cancer Survivors.” Translational Research 221: 65–82. 10.1016/j.trsl.2020.03.013. 32360946 PMC7321876

[acel70535-bib-0039] North, B. J. , and D. A. Sinclair . 2012. “The Intersection Between Aging and Cardiovascular Disease.” Circulation Research 110, no. 8: 1097–1108. 10.1161/CIRCRESAHA.111.246876. 22499900 PMC3366686

[acel70535-bib-0040] Noureddine, H. , G. Gary‐Bobo , M. Alifano , et al. 2011. “Pulmonary Artery Smooth Muscle Cell Senescence Is a Pathogenic Mechanism for Pulmonary Hypertension in Chronic Lung Disease.” Circulation Research 109, no. 5: 543–553. 10.1161/CIRCRESAHA.111.241299. 21719760 PMC3375237

[acel70535-bib-0041] Ohtani, N. 2022. “The Roles and Mechanisms of Senescence‐Associated Secretory Phenotype (SASP): Can It Be Controlled by Senolysis?” Inflammation and Regeneration 42, no. 1: 11. 10.1186/s41232-022-00197-8. 35365245 PMC8976373

[acel70535-bib-0042] Paradisi, G. , H. O. Steinberg , A. Hempfling , et al. 2001. “Polycystic Ovary Syndrome Is Associated With Endothelial Dysfunction.” Circulation 103, no. 10: 1410–1415. 10.1161/01.CIR.103.10.1410. 11245645

[acel70535-bib-0043] Parr, S. K. , J. Liang , K. L. Schadler , S. C. Gilchrist , C. C. Steele , and C. J. Ade . 2020. “Anticancer Therapy–Related Increases in Arterial Stiffness: A Systematic Review and Meta‐Analysis.” Journal of the American Heart Association 9, no. 14: e015598. 10.1161/JAHA.119.015598. 32648507 PMC7660726

[acel70535-bib-0044] Powe, C. E. , R. J. Levine , and S. A. Karumanchi . 2011. “Preeclampsia, a Disease of the Maternal Endothelium.” Circulation 123, no. 24: 2856–2869. 10.1161/CIRCULATIONAHA.109.853127. 21690502 PMC3148781

[acel70535-bib-0045] Powell, R. D. , J. H. Swet , K. L. Kennedy , et al. 2015. “MitoQ Modulates Oxidative Stress and Decreases Inflammation Following Hemorrhage.” Journal of Trauma and Acute Care Surgery 78, no. 3: 573–579. 10.1097/TA.0000000000000533. 25710429

[acel70535-bib-0046] Rawat, P. S. , A. Jaiswal , A. Khurana , J. S. Bhatti , and U. Navik . 2021. “Doxorubicin‐Induced Cardiotoxicity: An Update on the Molecular Mechanism and Novel Therapeutic Strategies for Effective Management.” Biomedicine & Pharmacotherapy 139: 111708. 10.1016/j.biopha.2021.111708. 34243633

[acel70535-bib-0047] Sato, E. , G. T. Feke , E. Y. Appelbaum , M. N. Menke , C. L. Trempe , and J. W. McMeel . 2006. “Association Between Systemic Arterial Stiffness and Age‐Related Macular Degeneration.” Graefe's Archive for Clinical and Experimental Ophthalmology 244, no. 8: 963–971. 10.1007/s00417-005-0201-6. 16411106

[acel70535-bib-0048] Saul, D. , R. L. Kosinsky , E. J. Atkinson , et al. 2022. “A New Gene Set Identifies Senescent Cells and Predicts Senescence‐Associated Pathways Across Tissues.” Nature Communications 13, no. 1: 4827. 10.1038/s41467-022-32552-1. PMC9381717 35974106

[acel70535-bib-0049] Shamoon, L. , J. A. Espitia‐Corredor , P. Dongil , et al. 2022. “Resolvin E1 Attenuates Doxorubicin‐Induced Endothelial Senescence by Modulating NLRP3 Inflammasome Activation.” Biochemical Pharmacology 201: 115078. 10.1016/j.bcp.2022.115078. 35551917

[acel70535-bib-0050] Tchkonia, T. , Y. Zhu , J. van Deursen , J. Campisi , and J. L. Kirkland . 2013. “Cellular Senescence and the Senescent Secretory Phenotype: Therapeutic Opportunities.” Journal of Clinical Investigation 123, no. 3: 966–972. 10.1172/JCI64098. 23454759 PMC3582125

[acel70535-bib-0051] Terwoord, J. D. , A. M. Beyer , and D. D. Gutterman . 2022. “Endothelial Dysfunction as a Complication of Anti‐Cancer Therapy.” Pharmacology & Therapeutics 237: 108116. 10.1016/j.pharmthera.2022.108116. 35063569 PMC9294076

[acel70535-bib-0052] Tian, X. , S. Chen , X. Xia , et al. 2024. “Causal Association of Arterial Stiffness With the Risk of Chronic Kidney Disease.” JACC Asia 4, no. 6: 444–453. 10.1016/j.jacasi.2023.10.010. 39100705 PMC11291385

[acel70535-bib-0053] Touil, Y. S. , N. Auzeil , F. Boulinguez , et al. 2011. “Fisetin Disposition and Metabolism in Mice: Identification of Geraldol as an Active Metabolite.” Biochemical Pharmacology 82, no. 11: 1731–1739. 10.1016/j.bcp.2011.07.097. 21840301

[acel70535-bib-0054] Venkatasubramanian, R. , M. A. Darrah , S. A. Mahoney , et al. 2025. “Cellular Senescence Mediates Doxorubicin Chemotherapy‐Induced Aortic Stiffening: Role of Glycation Stress.” Journal of Hypertension 82, no. 10: 1767–1777. 10.1161/HYPERTENSIONAHA.125.25408 PMC12327803 40747550

[acel70535-bib-0055] Venkatasubramanian, R. , S. A. Mahoney , D. A. Hutton , et al. 2025. “Cellular Senescence Mediates Doxorubicin Chemotherapy‐Induced Vascular Endothelial Dysfunction: Translational Evidence of Prevention With Senolytic Treatment.” American Journal of Physiology. Heart and Circulatory Physiology 329, no. 6: H1672–H1683. 10.1152/ajpheart.00712.2025. 41143747 PMC12704655

[acel70535-bib-0056] Venkatasubramanian, R. , S. A. Mahoney , D. A. Hutton , et al. 2025. “Cellular Senescence Mediates Doxorubicin Chemotherapy‐Induced Vascular Endothelial Dysfunction: Translational Evidence of Prevention With Senolytic Treatment.” *bioRxiv* 2025.04.19.649672. https://www.biorxiv.org/content/10.1101/2025.04.19.649672v1. 10.1152/ajpheart.00712.2025 PMC12704655 41143747

[acel70535-bib-0057] Wang, B. , J. Han , J. H. Elisseeff , and M. Demaria . 2024. “The Senescence‐Associated Secretory Phenotype and Its Physiological and Pathological Implications.” Nature Reviews. Molecular Cell Biology 25: 958–978. 10.1038/s41580-024-00727-x. 38654098

[acel70535-bib-0058] Wang, L. , D. Cao , H. Wu , H. Jia , C. Yang , and L. Zhang . 2019. “Fisetin Prolongs Therapy Window of Brain Ischemic Stroke Using Tissue Plasminogen Activator: A Double‐Blind Randomized Placebo‐Controlled Clinical Trial.” Clinical and Applied Thrombosis/Hemostasis 25: 107602961987135. 10.1177/1076029619871359. PMC6829632 31434498

[acel70535-bib-0059] Whitehead, J. C. , B. A. Hildebrand , M. Sun , et al. 2014. “A Clinical Frailty Index in Aging Mice: Comparisons With Frailty Index Data in Humans.” Journals of Gerontology. Series A, Biological Sciences and Medical Sciences 69, no. 6: 621–632. 10.1093/gerona/glt136. 24051346 PMC4022099

[acel70535-bib-0060] Yersal, Ö. , U. Eryilmaz , H. Akdam , N. Meydan , and S. Barutca . 2018. “Arterial Stiffness in Breast Cancer Patients Treated With Anthracycline and Trastuzumab‐Based Regimens.” Cardiology Research and Practice 2018: 5352914. 10.1155/2018/5352914. 29854434 PMC5954934

[acel70535-bib-0061] Yousefzadeh, M. J. , J. Zhao , C. Bukata , et al. 2020. “Tissue Specificity of Senescent Cell Accumulation During Physiologic and Accelerated Aging of Mice.” Aging Cell 19, no. 3: e13094. 10.1111/acel.13094. 31981461 PMC7059165

[acel70535-bib-0062] Yousefzadeh, M. J. , Y. Zhu , S. J. McGowan , et al. 2018. “Fisetin Is a Senotherapeutic That Extends Health and Lifespan.” eBioMedicine 36: 18–28. 10.1016/j.ebiom.2018.09.015. 30279143 PMC6197652

[acel70535-bib-0063] Zhu, Y. , E. J. Doornebal , T. Pirtskhalava , et al. 2017. “New Agents That Target Senescent Cells: The Flavone, Fisetin, and the BCL‐XL Inhibitors, A1331852 and A1155463.” Aging 9, no. 3: 955–963. 10.18632/aging.101202. 28273655 PMC5391241

