# Supplementary material for: Fisetin Supplementation Attenuates Premature Vascular Aging Induced by Doxorubicin via Suppression of Cellular Senescence and Mitochondrial Oxidative Stress
Source: Aging Cell. 2026 May 17;25(5):e70535. doi: 10.1111/acel.70535 (PMC13180696; doi:10.1111/acel.70535)
Supplement: Supplementary file 1 — Table S1: Primers. [file ACEL-25-e70535-s001.docx]

**SUPPLEMENTAL METHODS**

**Cell Culture Experiments.**

***Cell culture dosing paradigm.*** Human aortic endothelial cells (HAECs)(PromoCell; used at passage 3-4; female, age: 80 years, non-smoker, free from known CVD) were cultured in a 6-well glass bottom plate under standard culture conditions (37.5°C, 100% relative humidity, 5% CO2). HAECs were grown in basal media (Endothelial Cell Growth Medium-2 [EGM-2] BulletKit; PromoCell). HAECs were plated at 75,000 cells per well and treated with or without (control) 200nM doxorubicin (in DMSO) in EGM-2 for 24h(24). Following the initial incubation, media was replaced and doxorubicin-treated HAECs were treated with or without (control) increasing doses of fisetin (0.25, 0.5 and 1μM in DMSO) for 48h(23). Following second incubation, fresh media was replaced on all cells twice every 48h.

***Senescence-associated β-galactosidase (SA-β-gal) staining****.* SA-β-gal staining was performed using the Senescence Detection Kit (Abcam, Cambridge, UK, Cat. No. ab65351) according to the manufacturer’s instructions. Briefly, following cell culture dosing paradigm (above), HAECs were washed, fixed, incubated with the X-gal solution overnight, and stained with DAPI overnight. Images were captured using bright-field and fluorescent microscopy at 20X magnification and quantified using ImageJ, as described(25).

***Cell and Aortic Gene Expression****.* mRNA gene expression was measured in HAECs and segments of thoracic aorta following mechanical homogenization. RNA was extracted using the RNeasy mini kit (Qiagen, Hilden, Germany). cDNA was synthesized using the iScript cDNA synthesis kit (Bio-Rad Laboratories, Hercules, CA). Transcripts of cellular senescence and senescence-associated secretory phenotype (SASP) markers (primer sequences reported in **Table S1**) were analyzed using a StepOnePlus Real-Time PCR System (Applied Biosystems, Waltham, MA) in 96-well plates and the Taqman OpenArray (Applied Biosystems, Waltham, MA) was used as a master mix, as described(23). SimpleSeq DNA sequencing (Quintara Biosciences, Cambridge, MA) was used to validate PCR products.

**Animal Experiments.**

***Animals and Experimental Design****.* Male and female p16-3MR mice were bred and aged in our mouse colony at the University of Colorado Boulder. These mice carry a trimodal fusion protein (3MR) under the control of the p16^INK4A^ promoter which allows for selective genetic clearance of p16^INK4A^-positive senescent cells by administering the antiviral agent ganciclovir (GCV)(26). For the duration of the study, all mice were single housed at the University of Colorado Boulder animal facility with a 12hr:12hr light-dark cycle and allowed ad libitum access to an irradiated, fixed, and open rodent chow (Inotiv/Envigo 7917, stored at room temperature).

For the intervention, treatment groups were matched for baseline body weight and aortic PWV. At 4 months of age, mice were assigned to receive either a single intraperitoneal injection of Sham (sterile saline) or Doxo (10mg/kg in Sham). One week later, mice either received the vehicle (10% Ethanol, 30% PEG400 and 60% Phosal 50 PG) or fisetin (100mg/kg/day in vehicle). Treatment was administered via oral gavage using an intermittent dosing paradigm consisting of one week on treatment – two weeks off treatment – one week on treatment. Mice were sacrificed one to two weeks following the final dose to rule out any acute effects of the compound as the terminal half-life of fisetin is ~3.1 hours in plasma(27).

***Sacrifice and tissue collection****.* Mice were sacrificed using a method approved under the American Veterinary Medical Association guidelines. Mice were anesthetized under inhaled isoflurane anesthesia (open-drop method) and euthanized via cardiac exsanguination. The carotid arteries were immediately excised for endothelial function measurements. The heart, visceral adipose, quadricep, liver, and spleen was removed, cleaned, and weighed. The aorta was excised and rinsed in physiological saline solution (PSS), cleared of perivascular adipose tissue, and sectioned and stored as described below.

***Arterial Blood Pressure***. Systolic and diastolic blood pressures were assessed using a CODA noninvasive tail-cuff system (Kent Scientific, Torrington, CT), as we have described previously(9,23,28,29). Briefly, the pressure measurements from 20 collection cycles (following 5 acclimation cycles) for 3 consecutive days were averaged per mouse at each timepoint.

***Frailty.*** Frailty was assessed using a validated 31-point index covering 7 subdomains(30) that is similar and compares to clinical frailty indices in humans. Briefly, frailty was calculated and compared across age and treatment. A ‘0’ represents the absence of a deficiency, ‘0.5’ represents a mild deficit, and ‘1’ represents a severe deficit.

***Vascular endothelial function: endothelium-dependent dilation (EDD), cellular senescence-mediated suppression of EDD, NO-mediated EDD, mitochondrial-specific ROS suppression of EDD, and endothelium-independent dilation (EID)****.* Vasodilatory function was measured via *ex vivo* carotid artery EDD and EID in response to increasing doses of acetylcholine (ACh) and sodium nitroprusside (SNP), respectively, as described previously. In brief, after vessels were pre-constricted with phenylephrine (PE; 2mM; Sigma-Aldrich, Cat. No. P6126), EDD was assessed by measuring increases in luminal diameter in response to increasing concentrations of ACh (1X10^-9^ to 1X10^-4^ M; Sigma-Aldrich, Cat. No. A6625) with and without *ex vivo* coadministration of GCV (5μm, 180min pre-incubation; Sigma Aldrich, St. Louis, MO, Cat. No. G2536), mitochondrial-specific ROS scavenger MitoQ (1µM, 60min pre-incubation; Antipodean Pharmaceuticals, Inc., Menlo Park, CA, USA), or the NO synthase inhibitor *N*^G^-nitro-L-arginine methylester (L-NAME, 0.1mM, 30min pre-incubation; Sigma-Aldrich, Cat. No. N5751). NO-mediated dilation was then calculated as the difference between maximum EDD to ACh alone and in the presence of ACh and L-NAME:

NO-mediated dilation (%) = Maximum dilation_ACh_ - Maximum dilation_ACh+L-NAME_

Following EDD and pharamo-dissections of EDD, EID was assessed by measuring the increase in luminal diameter in response to increasing concentrations of SNP, an exogenous NO donor (1 X 10^-10^ to 1 X 10^-4^ M; Sigma-Aldrich, Cat. No. 13755-38-9). All dose response data are presented as percent dilation relative to maximum diameter to account for differences in baseline vessel diameter.

***Aortic mitochondrial ROS production****.* Aortic mitochondrial ROS production was assessed using the mitochondrial-specific spin probe 1-hydroxy-4-[2-triphenylphosphonio-acetamido]-2,2,6,6-tetramethylpiperidine (mitoTEMPO-H; Enzo Life Sciences, Cat. No. ALX-430-171-M005) by electron paramagnetic resonance (EPR) spectrometry, as previously described(9,23,28,29). In short, two 1-mm aortic rings were washed in warm physiological saline solution and incubated in Krebs/HEPES buffer, consisting of 99mM NaCl, 4.7mM KCl, 1.87mM CaCl_2_, 1.2mM MgSO_4_, 25mM NaHCO_3_, 1.03mM KH_2_PO_4_, 20mM Na-HEPES, 11.1mM glucose, 0.1mM diethylenetriaminepenta-acetic acid, 0.0035mM sodium diethyldithiocarbamate, and Chelex (Sigma-Aldrich, Cat. No. C7901), containing 0.5mM CMH or mitoTEMPO-H at 37°C for 60min. Samples were analyzed using aMS300 Xband EPR spectrometer (Magnettech, Berlin, Germany) with the following instrument parameters: B0-Field, 3350G; sweep, 80G; sweep time, 60s; modulation, 3000mG; MWatten, 7dB; gain, 500.

***In Vivo Aortic Stiffness****.* Aortic stiffness was assessed using the reference standard non-invasive *in vivo* measure, aortic PWV, one week before (pre) and one week after (post) the intervention, as previously described(9,29,31,32). Briefly, mice were placed under light isoflurane anesthesia (1.0-2.5%) and positioned supine on a warmed heat pad. Front- and hind-limb paws were then secured to corresponding ECG electrodes. Two Doppler probes were then placed on the skin at the transverse aortic arch and the abdominal aorta. Once clear R-waves were registered, three repeated 2-second ultrasound tracings were recorded and average pre-ejection time (*i.e.,* time between the R-wave of the ECG to the foot of the Doppler signal) was determined for each location. To calculate aortic PWV, the distance between the two probes was divided by the difference between the transverse aortic arch and abdominal aorta pre-ejection times (time_abdominal_ – time_arch_) and is reported as centimeters/second (cm/s).

***Aortic Intrinsic Mechanical Stiffness (Elastic Modulus) and Cellular Senescence-Mediated Changes in Aortic Intrinsic Mechanical Stiffness****.* Aortas were promptly excised from the mice following carotid artery excision, rinsed with cold physiological saline solution, and cleared of any remnant perivascular adipose and connective tissue. To measure *ex vivo* aortic stiffness, two thoracic aorta samples (~1mm in length) were cut and used to determine intrinsic mechanical stiffness via pin myography as we have previously described(9,23,28,29). In short, aorta samples were placed in heated (37°C) baths filled with calcium-free, phosphate-buffered saline (PBS). The samples were then mounted on two wire prongs, followed by three rounds of pre-stretching. Once pre-stretching was complete, aortic ring diameter was increased until 1mN of force was reached and incrementally increased by 5µm every 3min thereafter until failure. The force corresponding to each stretching interval was recorded and used to calculate stress and strain. A stress-strain curve was then generated using the following equations:

Strain (λ) = Δd/di

where *d* is diameter and *d_i_* is initial diameter;

Stress(t) = (λL)/2(HD)

where *L* is one-dimensional load, *H* is intima media thickness, and *D* is vessel length.

The elastic modulus of the stress-strain curve was determined as the slope of the linear regression fit to the final four points of the stress-strain curve, as previously reported by our laboratory (9,23,28,29). To assess the stiffening role of cellular senescence and the beneficial effects of fisetin on intrinsic mechanical stiffness, aortas from p16-3MR mice were pre-incubated with 5µM GCV, which are concentrations that have been shown to reduce the abundance of senescent cells *ex vivo*(24,28,33)*,* for 48h prior to the following measurements. Aortic intima media thickness and diameter were assessed as we have described previously(9,29,32). Briefly, aortic rings (1mm) were frozen in optimal cutting temperature solution and stored at -80°C until the time of sectioning. Aortic sectioning was performed on a cryostat (7µm; Leica CM300, Leica Biosystems, Wetzlar, Germany) at -22°C and sections were visualized, and images were captured with a bright-field microscope. Aortic intima media thickness and diameter were calculated using ImageJ software.

***Statistical Analyses****.* Power calculations were performed using G*power 3.1 (RRID: SCR_013726) for our primary outcome variable, aortic PWV. Previously, our laboratory has obtained effect sizes of 1.35 when comparing aortic PWV between treatment groups. With this effect size, N=6 mice per condition were required to achieve 99% statistical power. Additional mice were studied in each group to ensure sufficient PWV traces were obtained and to account for age-related attrition.

Statistical analyses were conducted using GraphPad Prism version 11.0 (GraphPad Software, Inc., San Diego, CA, USA; RRID:SCR_002798). Data were assessed for statistical outliers (ROUT test; Q=1%), and outliers were excluded from final analyses. Statistical significance was determined using one-way ANOVA. For experiments assessing the effects of two independent variables (e.g., pre/post-treatment or with/without incubation), a two-way ANOVA was performed. All ANOVAs were followed by Tukey’s post-hoc multiple comparisons tests to identify specific group differences. Statistical significance was set to α=0.05. Data are presented as mean ± SEM.

**SUPPLEMENTAL TABLES**

**Table S1. Primers.**

| **Gene** | **Species** | **Forward primer** | **Reverse primer** |
| --- | --- | --- | --- |
| *Cdkn2a* | Human | GAGCAGCATGGAGCCTTC | CCGTTTTCGACCCTGAGAG |
| *Cdkn1a* | Human | TCACTGTCTTGTACCCTTGTGC | TTTGCTCCTGTGCGGAAC |
| *Pai1* | Human | TCTGCCCTCACCAACATTCT | CGGTCATTCCCAGGTTCTCT |
| *Lmnb1* | Human | GAAAAAGACAACTCTCGTCGCA | GTAAGCACTGATTTCCATGTCCA |
| *Gapdh* | Human | ATGTTCGTCATGGGTGTGAA | GGTGCTAAGCAGTTGGTGGT |
| *Cdkn2a* | Mouse | CCCAACGCCCCGAACT | GCAGAAGAGCTGCTACGTGAA |
| *Cdkn1a* | Mouse | TTGCCAGCAGAATAAAAGGTG | TTTGCTCCTGTGCGGAAC |
| *Pai1* | Mouse | TGGAAGGGCAACATGACCAG | TCAGGCATGCCCAACTTCTC |
| *Lmnb1* | Mouse | GAGCCCCAAGAGCATCCAAT | CTGAGAAGGCTCTGCACTGT |
| *Tnf-α* | Mouse | ATGAGAAGTTCCCAAATGGC | CTCCACTTGGTGGTTTGCTA |
| *Vegf* | Mouse | AAAAACGAAAGCGCAAGAAA | TTTCTCCGCTCTGAACAAGG |
| *Ccl2* | Mouse | CACTCACCTGCTGCTACTCA | GCTTGGTGACAAAAACTACAGC |
| *Cxcl2* | Mouse | CCTGGTTCAGAAAATCATCCA | CTTCCGTTGAGGGACAGC |
| *Gapdh* | Mouse | AAGGTCATCCCAGAGCTGAA | CTGCTTCACCACCTTCTTGA |
